# Anchor clip–assisted detachable loop ligation for definitive hemostasis and closure of a high-risk bleeding gastric ulcer

**DOI:** 10.1055/a-2895-4120

**Published:** 2026-06-30

**Authors:** Zhongshang Sun, Miaomiao Li, Peng Shen, Qi Shi, Yuanyuan Xu, Shijie Ma

**Affiliations:** 1 Department of GastroenterologyThe Affiliated Huaian No.1 People's Hospital of Nanjing Medifrcal UniversityHuaian CityJiangsuChina

**Fig. 1:**
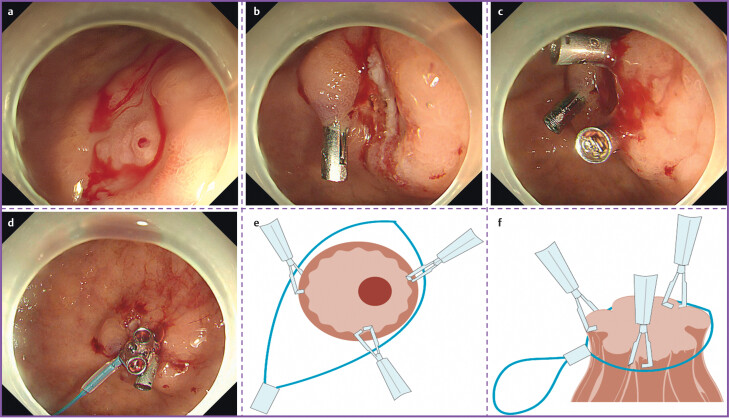
Anchor clip–assisted detachable loop ligation for a high-risk bleeding gastric ulcer. ( **a** ) An endoscopic view of a 2.0×2.0 cm ulcer on the lesser curvature of the gastric body with a red, adherent thrombotic plug and active oozing (high-risk stigmata). ( **b** ) The first anchor point established by deploying a hemoclip along the ulcer margin as preparation for anchor clip–assisted detachable loop ligation. ( **c** ) Three clips deployed circumferentially along the ulcer margin as anchor points. ( **d** ) A detachable loop placed around the anchors and progressively tightened, approximating the ulcer edges to achieve complete defect closure and mechanical ligation of the presumed culprit vessel. ( **e** ) Schematic illustration of three hemoclips deployed circumferentially along the ulcer margin as anchor points, with a detachable loop placed around the anchors. ( **f** ) Progressively tightened to approximate the ulcer margins, resulting in complete defect closure.

A 75-year-old man was admitted with a 2-week history of melena. Despite conservative medical treatment, including proton pump inhibitor therapy, he had ongoing evidence of active gastrointestinal bleeding and was referred for emergency endoscopic hemostasis. Endoscopy identified a 2.0×2.0 cm ulcer on the lesser curvature of the gastric body ( Fig. 1a ). A red, adherent thrombotic plug with active oozing was observed at the center of the ulcer base, consistent with a high-risk stigmata lesion. 1​ Initial hemostasis was achieved by thermal coagulation using hot biopsy forceps. Given the substantial risk of rebleeding, definitive mechanical closure and vessel control were pursued. Conventional through-the-scope clips may be limited by a relatively small opening angle and insufficient bite depth, which can hinder secure grasping of a broad, fibrotic ulcer base ( Fig. 1b ). Therefore, an anchor clip–assisted loop ligation technique was performed ( Video 1 ). Three hemoclips were deployed circumferentially along the ulcer margin to serve as anchor points ( Fig. 1c ). A nylon loop was then positioned around the anchors and progressively tightened, drawing the ulcer edges together to achieve complete defect closure and simultaneous mechanical ligation of the presumed culprit vessel ( Fig.1d–f ). Post-procedurally, the patient had no recurrent melena or signs of ongoing bleeding. He remained hemodynamically stable, required no further endoscopic intervention, and was discharged after clinical improvement.

**Video. 1** Anchor clip–assisted detachable loop ligation for definitive hemostasis and closure of a high-risk bleeding gastric ulcer.


Endoscopy_UCTN_Code_TTT_1AO_2AD
